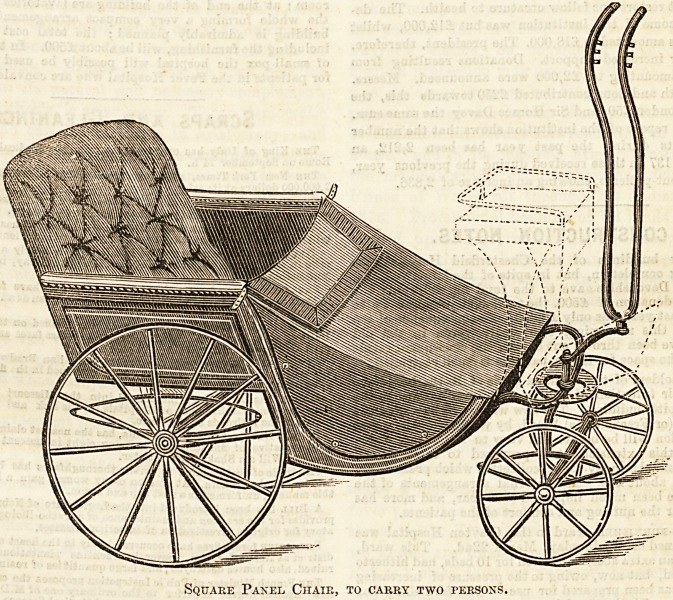# Invalid Chairs

**Published:** 1893-04-22

**Authors:** 


### PRACTICAL DEPARTMENTS.

### 1.—INVALID CHAIRS.

Immense improvements have been effected of late years in the construction of invalid chairs of all kinds. Messrs. Alderman and Johnson, Charing Cross Road, have every possible modern invention in this respect in stock, from the simple and inexpensive to the most elaborate and highly finished chairs and couches. Our illustration shows a portable carrying chair, made by Messrs. Alderman and Johnson, Charing Cross Road, which will be found invaluable in travelling, as it will fold into small and convenient compass, and can easily be stowed beneath the seat in a railway carriage. The handles can be adjusted to several positions, as may be required; a different elevation being fixed for carrying a patient up or down stairs, or for ordinary use on the level.

We saw, the other day, some very excellent carrying chairs of a less expensive kind than those described above, which will be within the reach of the many to whom such a convenience would be of the greatest comfort, but to whom the price of these highly finished chairs is absolutely prohibitive. These are made of bamboo and wicker, fitted with sliding handles, and sufficiently strong to bear considerable weight. They will be found very serviceable in many cases, though, of course, they are not suitable for very heavy patients.

In bath chairs there have been numerous improvements of late years, notably in those made of wicker, with self-guiding front wheels, and mounted on bicycle rubber-tyred wheels. These are so light in construction that they are no trouble to push, and can be guided without the least exertion on the part of the occupant.

Square Panel Chair, to carry two persons.

When the chair is merely needed for use in a room, the handles will revolve round to the side or fold under the seat, quite out of the way. It can be fitted with a seat cushion, which will make it the right height for a dining-room chair, a little point which will be duly appreciated by those who use it. This chair thus combines several advantages, and not the least of these is that it is very light in make, though at the same time thoroughly strong.

A more elaborate carrying chair, made by the above-mentioned firm, is provided with equilibrium handles, which, as the name implies, are self-adjusting to any angle, and can also be entirely removed if desired. There are many other varieties of this description of chair, some fitted with hand rails and adjustable foot rests; others made to separate from their stands, so that they can be lifted into a carriage and placed upon the seat. The stands are provided with automatic castors, and are therefore wheeled about with perfect ease.

We give an illustration of a pony chair, made to carry two persons, brought out by Mr. John Ward, 246, Tottenham Court Road. Many invalids will be glad to avail themselves of these useful miniature pony carriages, saving them the exertion of holding the reins, and enabling them to have a nurse or friend with them. The same maker has adopted an ingenious plan which largely reduces the difficulty experienced by semi-helpless invalids of getting in and out of the usual bath chair, owing to the height of the foot board from the ground. Mr. Ward obviates this trouble by so constructing the chair that it can be tilted from behind until the footboard touches the ground, rendering it an easy matter for the patient to step in and seat himself, when the attendant gently lowers the chair again upon the back wheel.

Of some of the many other newly invented and improved chairs and couches specially designed for cases of heart disease, &c., we propose to speak in our next article.

## Figures and Tables

**Figure f1:**